# Effects of hypoxia on human cancer cell line chemosensitivity

**DOI:** 10.1186/1471-2407-13-331

**Published:** 2013-07-05

**Authors:** Sara Strese, Mårten Fryknäs, Rolf Larsson, Joachim Gullbo

**Affiliations:** 1 Clinical Pharmacology, Department of Medical Sciences, Uppsala University, Akademiska Sjukhuset, 751 85 Uppsala, Sweden

**Keywords:** Chemotherapy, Hypoxia, Anoxia, Cancer cell lines, FMCA, Hypoxic incubator, Drug resistance

## Abstract

**Background:**

Environment inside even a small tumor is characterized by total (anoxia) or partial oxygen deprivation, (hypoxia). It has been shown that radiotherapy and some conventional chemotherapies may be less effective in hypoxia, and therefore it is important to investigate how different drugs act in different microenvironments. In this study we perform a large screening of the effects of 19 clinically used or experimental chemotherapeutic drugs on five different cell lines in conditions of normoxia, hypoxia and anoxia.

**Methods:**

A panel of 19 commercially available drugs: 5-fluorouracil, acriflavine, bortezomib, cisplatin, digitoxin, digoxin, docetaxel, doxorubicin, etoposide, gemcitabine, irinotecan, melphalan, mitomycin c, rapamycin, sorafenib, thalidomide, tirapazamine, topotecan and vincristine were tested for cytotoxic activity on the cancer cell lines A2780 (ovarian), ACHN (renal), MCF-7 (breast), H69 (SCLC) and U-937 (lymphoma). Parallel aliquots of the cells were grown at different oxygen pressures and after 72 hours of drug exposure viability was measured with the fluorometric microculture cytotoxicity assay (FMCA).

**Results:**

Sorafenib, irinotecan and docetaxel were in general more effective in an oxygenated environment, while cisplatin, mitomycin c and tirapazamine were more effective in a low oxygen environment. Surprisingly, hypoxia in H69 and MCF-7 cells mostly rendered higher drug sensitivity. In contrast ACHN appeared more sensitive to hypoxia, giving slower proliferating cells, and consequently, was more resistant to most drugs.

**Conclusions:**

A panel of standard cytotoxic agents was tested against five different human cancer cell lines cultivated at normoxic, hypoxic and anoxic conditions. Results show that impaired chemosensitivity is not universal, in contrast different cell lines behave different and some drugs appear even less effective in normoxia than hypoxia.

## Background

### Tumor hypoxia

Solid tumors contain regions with mild (hypoxia) to severe oxygen deficiency (anoxia), due to the lack of blood supply to the growing tumor nodules [1-3]. Oxygen and nutrients are essential for solid tumor growth, and when sufficient oxygen is not provided growth arrest or necrosis occurs in the unvascularized tumor core [4,5]. Neovascularization, or angiogenesis, is required to keep the growing tumor oxygenated and increased vascular density is correlated with increased metastasis and decreased patient survival in many cancers (reviewed by [6,7]).

Decreased oxygenation leads to various biochemical responses in the tumor cells that ultimately can result in either adaptation or cell death. Hypoxia-inducible factor α (HIF-1α) is one of the most important transcription factors and a regulator of gene products during hypoxia [8]. Initial or moderate increase of HIF-1α levels could lead to cell adaptation, and in the absence of oxygen cancer cells adjust to their new microenvironment mainly by angiogenesis stimulation by vascular endothelial growth factor (VEGF) [9], inhibition of apoptosis via Bcl-2 [10], modifying the cellular glucose/energy metabolism [11], adapting to acidic extracellular pH [12] and up-regulation of proteins involved in metastasis [13]. The delicate balance between activators and inhibitors regulate adaptation or cell death in growing tumor nodules.

### Hypoxia mediated resistance to radiotherapy and chemotherapy

Hypoxic cells may be resistant to both radiotherapy and conventional chemotherapy. Studies show that hypoxia has a negative impact of radiotherapy on tumor cells in various cancers such as mammary carcinoma [14], head and neck carcinoma [15] and uterine cervix carcinoma [16]. There are several non-excluding theories to explain the fact that also conventional chemotherapy has less effect on hypoxic tumor cells. The anarchic vascular pattern characteristic of many tumors includes caliber changes, loops and trifurcations [17]. This, and the distance between cell and blood vessel diminish the exposure of the anticancer drug and also the proliferation of the cells [4,18]. Since the cytotoxic effect is greater in rapidly dividing cells, the slow proliferating tumor cells far away from the blood vessels is less sensitive to chemotherapy [1,18]. Hypoxia also selects for cells with low expression of p53 and consequently p53-induced apoptosis is reduced in hypoxic cells [19]. In normoxic surroundings DNA injuries caused by some anticancer drugs is more permanent, while in hypoxic surroundings higher levels of restoration occurs [20]. Another association between hypoxia and chemotherapy resistance is the up-regulation of the multidrug resistance (MDR) genes and over expression of the gene product P-glycoprotein (P-gp), which is known to be involved in multidrug resistance [21,22].

Different methods have been applied to study the effect of a cytotoxic drug in an environment resembling that of a tumor, i.e. with tumor cells in a hypoxic environment. However, earlier in vitro studies on drug effects in hypoxic cells have been performed with different methods and have also yielded different results. For example, hypoxic or anoxic cells may be generated by incubation of monolayer cultures in hypoxic incubators with constant O_2_, N_2_ and CO_2_ concentrations [23-26], or by use of airtight containers, in which the oxygen concentration in the gas phase is held at a constant level, incubated in aerobic incubators [27]. The redox-potential in the medium can also be altered with, for example, cobalt chloride (CoCl_2_) to achieve chemical hypoxia [28] or enzyme generated oxygen depletion by adding glucose oxidase and catalase [29]. A three-dimensional way of studying the effect of drugs in hypoxia is the use of tumor spheroids [30,31]. Spheroids are generated by culturing adherent cells and give a 3D cellular context in which oxygen-, glucose- and ATP gradient varies [32]. After treatment, cell survival is measured to determine the relative hypoxic toxicity of a drug. This has previously been done by for example clonogenic [33] or non-clonogenic colorimetric assays using MTT [23,34,35], sulforhodamine B [36] or by trypan blue staining [24,26]. However, most of these investigations have been done with limited series of drugs and/or cell types, and slightly different conditions. In this work we have screened a larger panel of drugs in five different cell lines, to investigate their sensitivity to a panel of chemotherapeutic agents under conditions of normoxia (20% O_2_), hypoxia (1% O_2_), and anoxia (0.1% O_2_).

## Methods

### Cell lines

The in vitro analysis were carried out in a panel of cancer cell lines, including A2780 (ECACC Salisbury, UK), ACHN, MCF-7, NCI-H69 (all American Type Culture Collection, LGC Standards, Borås, Sweden) and U937-GTB (kind gift from Kennet Nilsson, Department of pathology, Uppsala University). The different cell lines were selected as representatives of various kinds of cancer types, including ovarian cancer (A2780), breast cancer (MCF-7), renal adenocarcinoma (ACHN), small cell lung cancer (H69) and a leukemic monocyte lymphoma (U937). Cell growth medium RPMI 1640 (Sigma-Aldrich, Stockholm, Sweden), supplemented with 10% heat-inactivated fetal bovine serum (FCS; Sigma-Aldrich, Stockholm, Sweden), 2 mmol/L L-glutamine, 100 μg/mL streptomycin, and 100 U/mL penicillin (Sigma-Aldrich, Stockholm, Sweden), was used to maintain A2780-, ACHN-, H69- and U937 cell lines. MCF-7 was maintained in Minimum Essential Medium Eagle (M5650, Sigma-Aldrich, Stockholm, Sweden), supplemented with 10% heat-inactivated FCS (Sigma-Aldrich, Stockholm, Sweden), 2 mmol/L L-glutamine, 100 *μ*g/mL streptomycin, 100 U/mL penicillin (Sigma-Aldrich, Stockholm, Sweden) and 1 mM sodium pyruvate (P5280, Sigma-Aldrich, Stockholm, Sweden). All cell lines were kept in 75 cm^2^ culture flasks (TPP, Trasadingen, Switzerland) at 37°C in a humidified atmosphere of 95% air, 5% CO_2_. The enzyme accutase (PAA, Pasching, Austria) was used to detach the A2780-, ACHN- and HT29 cells from the bottom of the flask and accumax (PAA, Pasching, Austria) was used to separate the H69 cells and detach the MCF-7 cells from the flask.

### Drugs and reagents

The drugs tested were selected as representatives of various chemotherapeutic drug groups with different modes of action. 5-fluorouracil (5-FU), cisplatin, docetaxel, doxorubicin, etoposide, gemcitabine, irinotecan, melphalan and vincristine were obtained from the Swedish Pharmacy (Uppsala Sweden). Acriflavine, digitoxin, digoxin, rapamycin, thalidomide and topotecan where purchased from Sigma-Aldrich (Stockholm, Sweden), mitomycin c from Medac (Varberg, Sweden), bortezomib and sorafenib from LC laboratories (Woburn, MA, USA) and tirapazamine from Chemos GmbH (Regenstauf, Germany). The drugs are listed in Table 1, including earlier reports of effect(s) in hypoxia. The pharmaceutical preparations were dissolved according to instructions from the manufacturer, the other drugs were dissolved in dimetylsulfoxid (DMSO; Sigma-Aldrich, Stockholm, Sweden) or dimethylacetamide (DMA; Sigma-Aldrich, Stockholm, Sweden) and stored frozen in −70°C for maximum three months. Sterile phosphate buffered saline (PBS; Sigma-Aldrich, Stockholm, Sweden) was used to dilute the drugs to desirable concentrations. Fluoresceindiacetate (FDA; Sigma-Aldrich, Stockholm, Sweden) was dissolved in DMSO to a concentration of 10 mg/mL and kept frozen (−20°C) as a stock solution protected from light.

**Table 1:** Drugs tested in this study, with previous reports of increased or decreased effect in hypoxia

**Drug**	**Type of drug**	**Effect in hypoxia**	**References**
5-FU	Antimetabolite pyrimidine analog	Less effective in hypoxia in mammary tumor and gastric cancer cell lines	[37,38]
Acriflavine	Antiseptic	Inhibition of HIF dimerization in a kidney cancer cell line	[39]
Bortezomib	Proteasome inhibitor	VEGF inhibitor in endothelial cells from myeloma patients, repress HIF-1α activity in multiple myeloma and liver cancer cell lines	[40,41]
Cisplatin	Platinum compound	Less effective in hypoxia in testicular germ cell tumor and gastric cancer cell lines	[35,38]
		Downregulate HIF in human ovarian cancer cell lines	[42]
Digitoxin	Cardiac glycoside	HIF-1 inhibition in hepatoblastoma cell line	[43]
Digoxin	Cardiac glycoside	HIF-1 inhibition in prostate cancer, hepatoblastoma and lymphoma cell lines	[43]
Docetaxel	Mitosis inhibitor, taxane	HIF-1 inhibition in ovarian and breast cancer cell lines	[44]
		Activity unchanged in prostate and ovarian cancer cell lines	[42,45]
Doxorubicin	Antracycline, topoisomerase II inhibitor	Inhibition of HIF activation in human ovarian cancer cell lines	[42]
		Less effective in hypoxia in murine sarcoma cell lines	[46]
Etoposide	Mitosis inhibitor, epipodo-phyllotoxin	Less effective in hypoxia in testicular germ cell tumor, breast, prostatic and hepatic cell lines	[35,47,48]
Gemcitabine	Pyrimidine analog	Less effective in hypoxia in testicular germ cell tumor and pancreatic cell lines	[35,49,50]
Irinotecan	Topoisomerase I inhibitor	The metabolite SN38 inhibits HIF-1α and VEGF in glioma cell lines	[51]
Melphalan	Alkylating mustard analog	Enhanced effect in hypoxia in an animal model and in multiple myeloma cell lines	[52,53]
Mitomycin c	Quinone antibiotics	Bioreductive in hypoxia in murine sarcoma and mammary cell lines	[46,54]
		Less effective in hypoxia in testicular germ cell tumor cell lines	[35]
Rapamycin	Oral macrolide, mTOR-inhibitor	Inhibits mTOR, downregulate VEGF, degrades HIF-1 in prostate cancer, hematopoietic and colon cancer cell lines	[55-57]
Sorafenib	Multikinase inhibitor	VEGFR and PDGFR inhibitor in hepatocellular carcinoma	[58]
Thalidomide	Anti-inflammatory	Angiogenesis inhibitor in CAM-assay and human endothelial cells	[59,60]
Tirapazamine	Bioreductive prodrug	Reactive radical cause DNA- breaks in several hypoxic human and animal cell lines	[61-63]
Topotecan	Topoisomerase I inhibitor	Inhibit HIF-1α expression in glioblastoma cell lines and tumor biopsies	[64,65]
Vincristine	Vinca alkaloid	Inhibit HIF-1α expression in ovarian and breast cancer cell lines	[44]
		Less effective in hypoxia in gastric cancer cells	[66]

### Oxygen deprivation

The cells were seeded in duplicate in 96-well microtiter plates (NUNC, Roskilde, Denmark). 180 μL cell suspension, with the concentration of 100 000 cells/mL was added to each well, blank wells containing medium only. The normoxic set of plates was placed in an aerobic incubator (atmospheric) and the hypoxic/anoxic set where moved to a Ruskinn InVivo_2_ 500 hypoxic incubator (Ruskinn Technology Ltd, Pencoed, UK) and where equilibrated at 37°C in a humidified atmosphere of 5% CO_2_ and limited oxygen, either 0.1% O_2_ or 1.0% O_2_. Hereafter 0.1% O_2_ is considered as extreme deprivation of oxygen and will be referred to as anoxia and 1.0% O_2_ will be referred to as hypoxia. After 18 hours pre-incubation, 20 μL of test solution were added to each well (PBS to blank and control, drug solution to duplicate test wells) and left to incubate for 72 hours. After the incubation, measurement according to the fluorometric microculture cytotoxicity assay (FMCA) was performed.

### The Fluorometric Microculture Cytotoxicity Assay FMCA

The non-clonogenic cell viability assay FMCA is based on the fluorescence generated from the hydrolysis of fluoresceindiacetate (FDA) to fluorescein by cells with intact cell membranes. The methodology is described by Larsson et al. (1992) and also in detail in the protocol article by Lindhagen et al. (2008) [67,68]. In short, cells (20000/well) were pre-incubated at normoxia, hypoxia or anoxia, where after drugs were added and the plates incubated for 72 hrs, washed ones with PBS in a microtiter plate washer (Multiwash, Dynatech Laboratories) and thereafter FDA (100 μl of 0.01 mg/mL FDA, Sigma-Aldrich, Stockholm, Sweden) in a buffer, was added. After 40 minutes incubation (37°C) the generated fluorescence was measured at 485/520 nm in a Fluoroskan II (Labsystems, Helsinki Oy, Finland) and the survival index (SI%) for each drug concentration was calculated. All experiments were performed three times. From the mean SI%-curves the half maximal inhibitory concentration (IC_50_) was determined using non-linear regression analysis in Prism 5 Software Package (Graph Pad, San Diego, CA). Cytotoxicity ratios (R_anox_ = anoxic IC_50_/normoxic IC_50_ and R_hypox_ = hypoxic IC_50_/normoxic IC_50_) were determined for each drug and cell line.

### Statistical analysis

For the three obtained SI% replicates, Grubbs test was used to detect and exclude significant outliers, with the significance level of alpha = 0.05. Calculations of IC_50_ were made by the non-linear regression analysis in the Prism 5 software. If the IC_50_ was ambiguous it was reported as not applicable (N/A). If the suggested IC_50_ exceeded the highest tested concentration it was reported only if the R^2^ exceeded 0.75 or SI% for the highest concentration was under 75%, otherwise only defined as > highest tested concentration. An approximate (~) value was used as a true value when used to calculate cytotoxicity ratios. An unpaired two-tailed *t*-test was used to determine the significance levels of the ratios (p < 0.05, p < 0.01 and p < 0.001).

### Verifying hypoxia

To verify hypoxia and anoxia in the cells, microarray analysis was performed as previously described [69] at the Uppsala Array Platform (Department of Medical Science, Science for Life Laboratory, Uppsala University, Sweden). MCF-7 breast cancer cells was incubated either in normoxic, hypoxic or anoxic surroundings, after 90 hours the cells were washed with PBS and total RNA was prepared using RNeasy® Mini Kit (Qiagen AB, Sollentuna, Sweden) according to the manufacturers instructions. RNA concentration was measured with ND-1000 spectrophotometer (NanoDrop Technologies, Wilmington, DE) and RNA quality was evaluated using the Agilent 2100 Bioanalyzer system (Agilent Technologies Inc, Palo Alto, CA). 250 ng of total RNA from each sample were used to generate amplified and biotinylated sense-strand cDNA from the entire expressed genome according to the Ambion WT Expression Kit (P/N 4425209 Rev C 09/2009) and Affymetrix GeneChip® WT Terminal Labeling and Hybridization User Manual (P/N 702808 Rev. 6, Affymetrix Inc., Santa Clara, CA). GeneChip® ST Arrays (GeneChip® Human Gene 2.0 ST Array) were hybridized for 16 hours in a 45°C incubator, rotated at 60 rpm. According to the GeneChip® Expression Wash, Stain and Scan Manual (PN 702731 Rev 3, Affymetrix Inc., Santa Clara, CA) the arrays were then washed and stained using the Fluidics Station 450 and finally scanned using the GeneChip® Scanner 3000 7G. The raw data was normalized in the free software Expression Console provided by Affymetrix (affymetrix.com) using the robust multi-array average (RMA) method. Further interpretation of the gene expression data was done by gene set enrichment analysis (GSEA) [70] and the gene ontology (GO) bioinformatic tool: database for annotation, visualization and integrated discovery (DAVID) [71].

## Results

The normoxic IC_50_-values for all drugs in the panel in the cell lines (A2780, ACHN, H69, MCF-7 and U-937) are shown in Table 2 and the IC_50_-ratios of hypoxic or anoxic vs normoxic cells are displayed in Table 3. If the ratio for a drug was close to 1 (arbitrarily set to 0.8-1.2), it was considered as equally effective in anoxic/hypoxic and normoxic cells. If the ratio exceeded 1.2 the effect of the drug was less effective in anoxia/hypoxia, and if the ratio was less than 0.8 the drug was more effective in anoxia/hypoxia.

**Table 2:** Mean IC_50_ values for all tested drugs in normoxia A2780, ACHN, H69, MCF-7 and U-937

	**A2780**	**ACHN**	**H69**	**MCF-7**	**U-937**
**5-FU**	0.69 mM	>1.0 mM	>1.0 mM	N/A	0.14 mM
**Acriflavine**	6.2 μM	12 μM	27 μM	61 μM	4.6 μM
**Bortezomib**	11 nM	0.63 μM	15 nM	>3.0 μM	13 nM
**Cisplatin**	~9.3 μM	28 μM	0.11 mM	62 μM	2.8 μM
**Digitoxin**	0.11 μM	0.15 μM	>20 μM	N/A	70 nM
**Digoxin**	0.15 μM	0.81 μM	N/A	N/A	0.12 μM
**Docetaxel**	10 μM	4.0 μM	9.5 μM	25 μM	<16 nM
**Doxorubicin**	~1.8 μM	4.7 μM	23 μM	29 μM	0.15 μM
**Etoposide**	34 μM	11 μM	17 μM	>50 μM	0.17 μM
**Gemcitabine**	~4.8 mM	>5.0 mM	>5.0 mM	>5.0 mM	<1.6 μM
**Irinotecan**	38 μM	17 μM	~0.21 mM	93 μM	4.7 μM
**Melphalan**	18 μM	37 μM	0.13 mM	0.12 mM	6.0 μM
**Mitomycin C**	~53 μM	23 μM	37 μM	35 μM	0.70 μM
**Rapamycin**	~47 μM	28 μM	62 μM	N/A	5.1 μM
**Sorafenib**	7.2 μM	15 μM	~13 μM	43 μM	7.3 μM
**Thalidomide**	N/A	>0.1 mM	>0.1 mM	>0.1 mM	0.17 mM
**Tirapazamine**	0.15 mM	64 μM	0.15 mM	0.14 mM	24 μM
**Topotecan**	15 μM	10 μM	9.5 μM	<30 μM	0.12 μM
**Vincristine**	3.5 mM	<0.1 mM	N/A	N/A	<34 nM

N/A (not applicable) refers to an ambiguous IC_50_.

**Table 3:** Ratios R_anox_ and R_hypox_ for all tested drugs in all cell lines

	**A2780**		**ACHN**		**H69**		**MCF-7**		**U-937**	
	**Anoxia**	**Hypoxia**	**Anoxia**	**Hypoxia**	**Anoxia**	**Hypoxia**	**Anoxia**	**Hypoxia**	**Anoxia**	**Hypoxia**
**5-FU**	0.29***	N/A	N/A	N/A	N/A	N/A	N/A	N/A	1.7**	1.5
**Acriflavine**	0.56	N/A	2.4*	1.0	0.19**	0.23**	N/A	0.52	0.99	0.81*
**Bortezomib**	0.10***	0.20***	>4.0**	>6.0**	0.14**	0.76	N/A	N/A	1.1	0.85
**Cisplatin**	1.6	0.90	3.6**	1.1	0.22**	0.21**	0.37***	0.49**	0.52***	0.43***
**Digitoxin**	3.6	0.94	N/A	>133**	<0.050	<0.56	N/A	N/A	2.0	0.95
**Digoxin**	2.4	1.1	N/A	>80**	N/A	<0.24	N/A	N/A	1.2	0.85
**Docetaxel**	2.3	1.0	44***	7.3**	1.0	1.8	0.75	0.58	N/A	N/A
**Doxorubicin**	1.3	0.50	3.5**	1.1	1.1	<0.46	>2.1*	0.59*	0.69*	0.61**
**Etoposide**	1.3	0.73	>3.7**	>6.5**	0.46*	0.75	N/A	N/A	0.87	0.80*
**Gemcitabine**	N/A	0.62	N/A	N/A	N/A	N/A	N/A	N/A	N/A	N/A
**Irinotecan**	1.3	1.3	5.3***	>2.7**	0.32	N/A	>2.7	N/A	1.1	0.86
**Melphalan**	2.0	2.6	4.9***	5.1**	0.24**	N/A	0.89	N/A	0.61**	0.55**
**Mitomycin C**	0.17***	0.22	1.1	2.0**	0.11***	0.18**	0.63*	0.31***	0.45***	0.44***
**Rapamycin**	1.2	0.34	1.2	1.3	0.14**	0.08**	N/A	N/A	0.34*	1.8
**Sorafenib**	0.85	0.87	1.6*	1.2	0.58	1.8	1.5	1.4	2.0	1.2
**Thalidomide**	N/A	N/A	N/A	N/A	<0.89*	N/A	<0.28	N/A	0.21***	<0.44*
**Tirapazamine**	0.044***	0.045***	0.1***	0.10***	0.025***	0.042***	0.055***	0.062***	0.072***	0.094***
**Topotecan**	0.023***	0.50	N/A	1.5	N/A	0.31**	N/A	N/A	2.2*	0.72
**Vincristine**	5.0**	0.010	N/A	N/A	N/A	N/A	N/A	N/A	N/A	>1

R_anox_ = anoxic IC_50_/normoxic IC_50_ and R_hypox_ = hypoxic IC_50_/normoxic IC_50_. Ratio value for a drug: 0.8-1.2 = equally effective in anoxia/hypoxia and normoxia, <0.8 = more effective in anoxia/hypoxia, >1.2 = more effective in normoxia, N/A = not applicable. Significance levels * p < 0.05; ** p < 0.01; *** p < 0.001; two-tailed *t*-test.

### Trends in the different cell lines

The ovarian carcinoma cell line A2780 was less sensitive to most drugs (ratio >1.2 in nine of 17 drugs evaluable for IC_50_) in anoxia (0.1% O_2_), but surprisingly was more or equally sensitive (ratio <1.2 in 14 of 16 drugs) to the administered drugs in hypoxia (1.0% O_2_) compared to normoxia. The renal adenocarcinoma ACHN was less sensitive to the effects of most drugs in both anoxic (ratio >1.2 in 10 of 12 drugs) and hypoxic cells (ratio >1.2 in 11 of 15 drugs) compared to normoxic cells. Compared to normoxic cells, oxygen deprived H69 (small lung cancer) and MCF-7 (breast cancer) cells were generally more sensitive to most drugs (for hypoxia the ratio was <0.8 in 11 of 13, and in 6 of 7 drugs respectively). U-937 (lymphoma) cells were slightly more, or equally, sensitive to most drugs in a hypoxic environment.

### Trends between the different drugs

**Figure 1:**
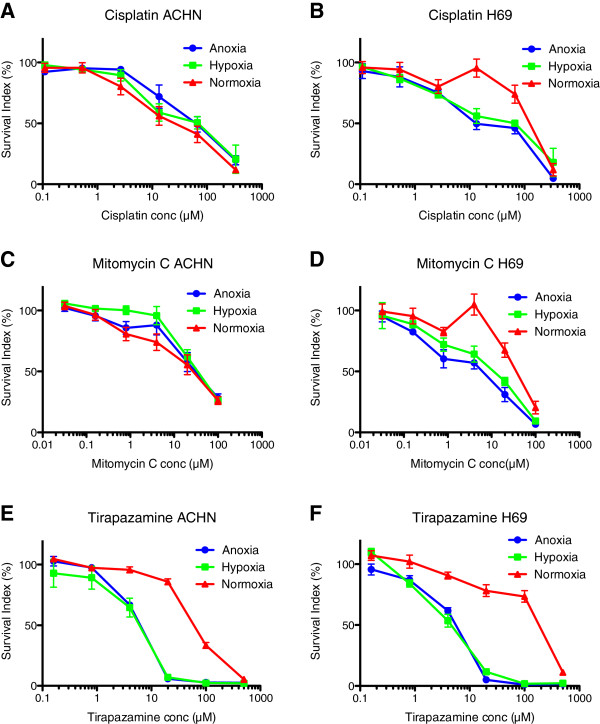
**The effect of drugs generally more effective in oxygen deprived environment.** Cisplatin **(A** and **B)**, mitomycin c **(C** and **D)** and tirapazamine **(E** and **F)** in ACHN (renal adenocarcinoma) and H69 (small lung cancer) cell lines in anoxic, hypoxic and normoxic surroundings. Error bars denote SEM.

**Figure 2:**
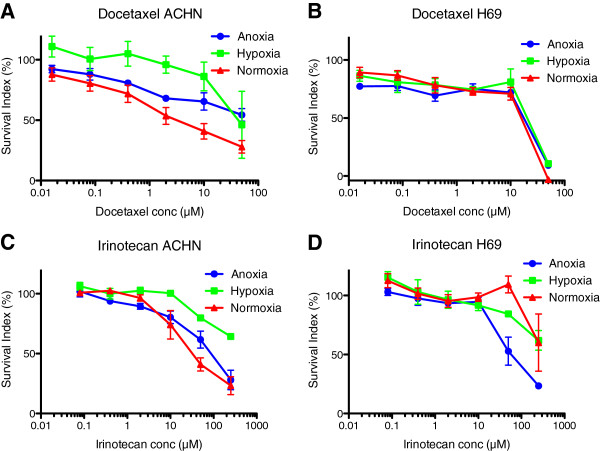
**The effect of drugs generally less effective in oxygen deprived environment.** Docetaxel **(A** and **B)** and irinotecan **(C** and **D)** in ACHN (renal adenocarcinoma) and H69 (small lung cancer) cell lines in anoxic, hypoxic and normoxic surroundings. Error bars denote SEM.

In general cisplatin, mitomycin c and tirapazamine (Figure 1) were more effective in anoxic or hypoxic environment (e.g. tirapazamine was significantly more active in all evaluated cell lines; cisplatin in H69, MCF-7 and U-937; and mitomycin C in A2780, H69, MCF-7 and U-937). Acriflavine, bortezomib, doxorubicin and etoposide also showed a slightly higher effect in anoxia and hypoxia compared to normoxia. Sorafenib and irinotecan (Figure 2) was apparently less effective in most anoxic and hypoxic cells (e.g. sorafenib was significantly less active in ACHN, MCF-7 and U-937), while docetaxel and melphalan had a slight decrease in effect in most anoxic and hypoxic cells. The other tested drug did not present with a clear tendency for being more or less sensitive in hypoxia or anoxia, the different cell types behaved differently (Table 3).

### Sensitivity of untreated cells

The control/blank signal relationship between oxygen deprived and oxygenated cells were calculated to evaluate the proliferating abilities of the cells, since a diminished proliferative capacity is likely to render lower sensitivity to most cytotoxic drugs. The mean ratio of the control/blank signal of anoxic or hypoxic cells and normoxic cells are presented in Table 4. A value below 1 (a lower signal) indicates a lower cell number in control wells after 18 + 72 hrs incubation in oxygen deprived cells vs. normoxic cells, as would be expected theoretically. However, the comparably high cell density (100,000/mL, selected to give the best signal-noise ratio) and the 90 hrs total incubation will probably also lead to some extent of growth inhibition due to confluence and cell-cell inhibition in the normoxic cells during the experiment. In such cases it is possible that growth inhibition (i.e. cytostatic effects, in contrast to cell killing cytotoxic effects) in the end of the experiments may be underestimated. Low ratios were observed in ACHN, U-937 and anoxic A2780 cells, which appear to correlate with the lower sensitivity to most drugs in hypoxic/anoxic ACHN and anoxic A2780 cells. However, it appears that U-937 is the most sensitive cell line to oxygen deprivation in the panel, and this is not reflected by the changes in chemosensitivity. Surprisingly, a high ratio was observed in H69, and indeed this cell line was also generally more sensitive to most of the drugs tested. No significant discrepancy was observed in MCF-7, who still was slightly more sensitive to the drugs in hypoxia.

**Table 4:** Mean ratio of the control/blank signal in cells cultivated under anoxic/hypoxic condition vs normoxia (n = 6)

	**Hypoxia**	**Anoxia**
**A2780**	0.90	0.76*
**ACHN**	0.78*	0.68*
**H69**	1.29*	1.23
**MCF-7**	0.94	0.97
**U-937**	0.57**	0.53**

Ratio value ~1 = equal cell number at 90 h in anoxia/hypoxia or normoxia, <0.8 = lower cell number in anoxia or hypoxia, >1.2 = higher cell number in anoxia/hypoxia. Significance levels * p < 0.05; ** p < 0.01; paired, two-tailed *t*-test.

### Hypoxia verification

**Figure 3:**
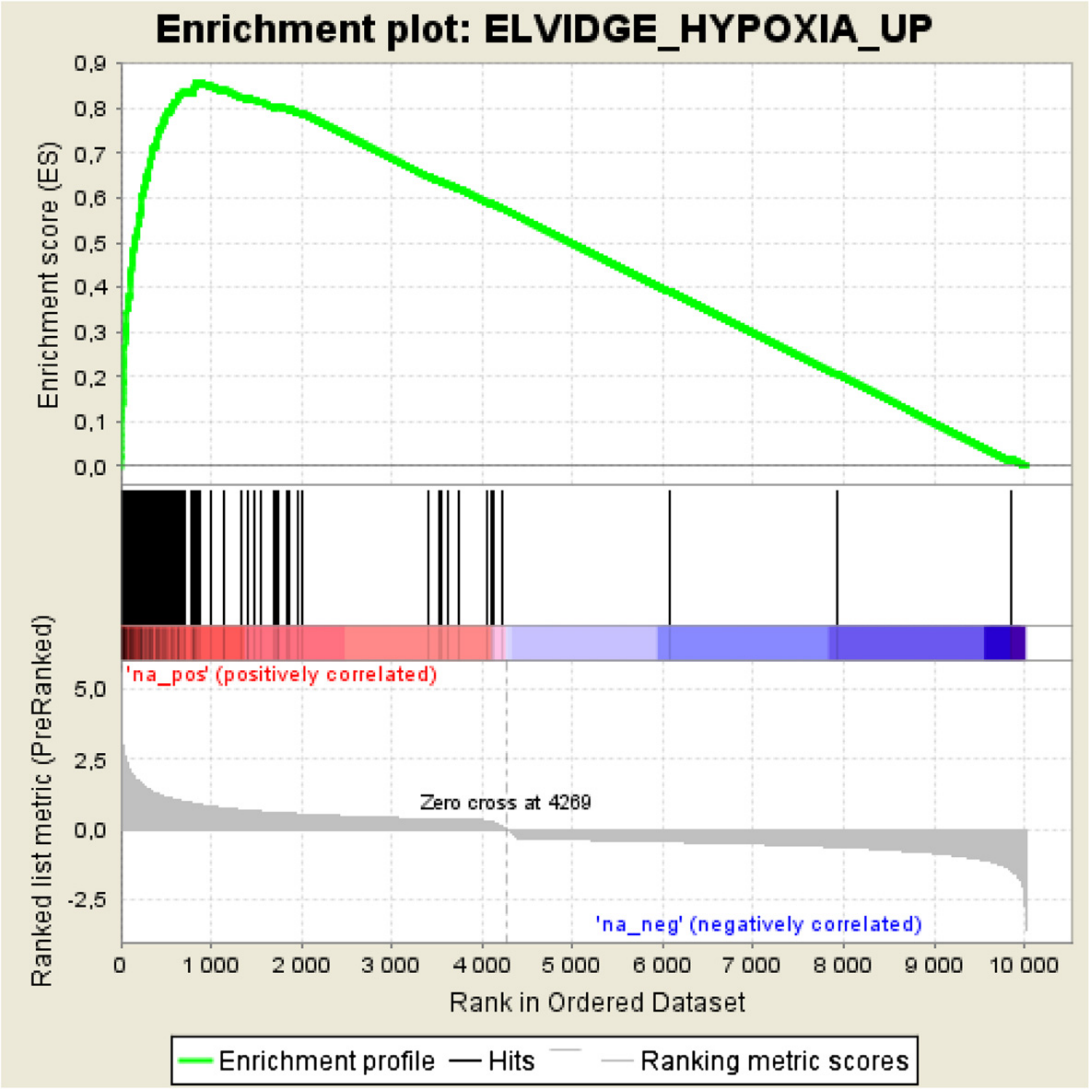
**Gene set enrichment analysis.** Results based on gene expression data from breast cancer cells (MCF-7 cell line) incubated in hypoxia (1.0% O_2_) compared to normoxia (20% O_2_) for 90 hrs. Enrichment profile shows an association of hypoxia-associated genes among the genes up-regulated when incubated in hypoxia.

Gene set enrichment analysis shows a distinct pattern of hypoxia-associated gene sets among the genes up-regulated when incubated in hypoxia [72]. Gene expression data confirmed that cells grown in oxygen-deprived surroundings to a higher degree expressed genes affiliated with hypoxia such as HIF1α (Figure 3). A clear pattern was also seen in the over-represented GO terms with the top result being the “response to hypoxia” (adjusted p-value 5.19E-13) group of 16 genes, also for the up-regulated genes. Raw and normalized expression data have been deposited at Gene Expression Omnibus with accession number GSE47009.

## Discussion

The concentration of oxygen in human tumors widely varies, and it is not uncommon to find areas with oxygen pressure lower than 2.5 mmHg, and the extent of hypoxia seems to be tumor stage and size independent [73]. Radiotherapy and conventional chemotherapies are often less effective in oxygen depressed cells [74]. Therefore it is of great importance to make use of the oxygen deprivation and find drugs that are more effective in hypoxic tumor cells.

In our study the untreated hypoxic and anoxic ACHN and U-937 cells, as well as anoxic A2780 cells were less proliferative than corresponding normoxic cells (i.e. most sensitive to oxygen deprivation). Indeed results also showed that ACHN and anoxic A2780 were more resistant to most drugs under reduced oxygen pressure, which is expected in view of the fact that slow proliferating tumor cells are less sensitive to chemotherapy. Interestingly the reversed effect could be observed in H69, where oxygen deprived cells (most surprisingly) appeared more viable and was a lot more sensitive to drugs. MCF-7 cells were also more sensitive to drugs in an oxygen-deprived environment but, in difference to H69, the MCF-7 cells displayed no proliferative difference in normoxic and hypoxic or anoxic surroundings. Hypoxia mostly occurs in tumors and therefore different cell lines with a solid tumor origin were the most interesting objects in this study. The leukemic lymphoma cell line U-937 is not a solid tumor per se, but was included in the study for comparison. Untreated U-937 cells were less viable in an oxygen-deprived environment, but did not display any real difference in sensitivity to chemotherapy in hypoxia or anoxia.

Three drugs were more effective in a hypoxic and anoxic environment; cisplatin, mitomycin c and tirapazamine. Earlier studies have revealed contradictive results, showing hypoxic cells to be more resistant to cisplatin in some cell lines [35] but also showing cisplatin to be a HIF-1 inhibitor [42]. Mitomycin c was also clearly more effective in most of the oxygen deprived cell lines. Hypoxia induces the enzymatic system capable of activating mitomycin c [75] and is therefore considered more toxic to hypoxic cells [46,54]. However, mitomycin c has also been shown to be less effective in hypoxic testicular germ cell tumor cell lines [35] and was in our study less effective in ACHN under hypoxic and anoxic conditions. Tirapazamine was significantly more effective in all oxygen deprived cell lines, and our results for tirapazamine highly correspond to previous studies of this bioreductive prodrug [62]. Tirapazamine is activated under hypoxic conditions by a reductase enzyme, in which creating a highly reactive molecule that in turn causes single- and double strand breaks in the DNA of tumor [61].

The drugs with increased resistance in hypoxic and anoxic cells were docetaxel, irinotecan, melphalan and sorafenib. Docetaxel has been shown to both influence [44] and not influence [42] the HIF-1α protein accumulation. Although this study proposed that docetaxel was associated with increased drug resistance in most cells in anoxia and hypoxia, other studies has implied that some cell lines was not [45]. In accordance to this study, irinotecan has earlier been shown to be less effective under hypoxic conditions [35]. Irinotecan decreases the expression of HIF-1α and VEGF under both normoxic and hypoxic conditions [51], which could be why there is no difference in effect in some cell lines; here U-937. Melphalan is an alkylating agent with an enhanced effect in hypoxia [52] and in HIF-1α inhibited cells [53]. Although the correlation between hypoxia and melphalan resistance was not distinct, both A2780 and ACHN were clearly less sensitive and U-937 more sensitive, in oxygen deprived cells. Sorafenib inhibits vascular endothelial growth factor receptor (VEGFR) and platelet-derived growth factor receptor (PDGFR) signaling [58], thus one might hypothesize that sorafenib would be more potent under hypoxic conditions. With respect to the cell lines used in this report, we have found no information on SCLC cell line NCI-H69 expression or dependence on VEGF signaling. The renal cell adenocarcinoma ACHN has a low normal baseline secretion of VEGF to cell growth medium [76], a secretion that may be inhibited by sorafenib, and to which ACHN is sensitive [77]. The breast cancer cell line MCF-7 has been described with a survival system by which VEGF can act as an internal autocrine (intracrine) survival factor through its binding to VEGFR-1 [78], and cell line is sensitive to treatment with sorafenib, which also appear to down-regulate hypoxia induced HIF-1α expression [79]. The ovarian carcinoma cell line A2780 expresses VEGFR-1 [80], but its sensitivity to sorafenib has not been described previously. In this study sorafenib was less effective in hypoxic and anoxic ACHN, MCF-7 and U-937 cells, which may be related to the mono-culture assay with no communicating stroma cells.

In the study presented herein we have emphasized to isolate hypoxia as the variable in the experiments, all other factors (nutrients in medium, cell density, incubation time etc.) were standardized, and all arms of each replicate (normoxic vs anoxic/hypoxic) were analyzed simultaneously. There are several environmental factors in solid tumors that may be studied, e.g. the low nutrient supply (analogous with oxygen supply), interaction with stroma cells, acidity (in part secondary to hypoxia, and metabolism), as well as proliferation of the tumor cells. These factors may be studied individually (as in this report), or by assays including several aspects, for example by the use of spheroid cultures or prolonged incubation times beyond confluency. Furthermore, since different drugs act on cancer cells in different ways resulting in cytostatic (growth inhibitory) or cytotoxic (cell killing) effects, different readouts would probably yield different results. The FMCA-based IC_50_-value used in this report is based on survival indices (compared to untreated control) at the end of the experiment, and is thus the result of both antiproliferative and toxic effects.

## Conclusion

Our results show that impaired chemosensitivity is not universal, in contrast different cell lines behave different and some drugs appear even less effective in normoxia. Part of the results obtained with this method, as probably with any model of oxygen deficiency, can be directly explained by decreased proliferation when cells are deprived of oxygen. However, this is clearly not the only variable, as some cells appeared to increase their proliferation and sensitivity under low oxygen pressure. Furthermore, hypoxia is not the only limiting factor of proliferation in a small tumor, but other limiting factors, such as the physical space, distribution of nutrients and drugs, metabolism and removal of waste products (with a succeeding change in pH), may also be utilized as therapeutic targets. These and other factors could also be evaluated in a similar screen study.

## Competing interests

The authors declare that they have no competing interests.

## Authors’ contributions

SS individually performed all experimental work, collected and processed raw data, and drafted the manuscript. MF is appointed co-supervisor, participated in the experimental design and in particular interpretation of microarray analysis. RL is appointed co-supervisor and head of department, RL participated in the design of the study and interpretation of data. JG is appointed main supervisor, conceived of the study, participated in its design and co-ordination with co-workers/authors. JG was also involved in analyzing and interpretation data yielded, and had an active role in drafting the manuscript together with SS. All authors read and approved the final manuscript.

## Pre-publication history

The pre-publication history for this paper can be accessed here:

http://www.biomedcentral.com/1471-2407/13/331/prepub
